# Stem cell–based strategies for HIV-1 remission: Emerging frontiers and translational challenges

**Published:** 2025

**Authors:** Giovannino Silvestri, Aditi Chatterjee

**Affiliations:** 1 Marlene and Stewart Greenebaum Comprehensive Cancer Center, Baltimore, MD 21201, USA; 2 Department of Medicine, University of Maryland School of Medicine, Baltimore, MD 21201, USA; 3 Member Cancer Therapeutics Program

## Introduction

Antiretroviral therapy (ART) has transformed HIV-1 from a fatal infection into a manageable chronic condition. Yet ART cannot eradicate latent viral reservoirs, necessitating lifelong adherence and leaving more than 38 million people worldwide without a definitive cure [1]. In recent years, stem-cell–based strategies have gained prominence as potential curative approaches, particularly those inspired by the *CCR5Δ32* transplantation cases that provided proof-of-concept for long-term viral remission in the absence of ART.

The following sections synthesize lessons from transplantation, advances in gene editing, integration with immunotherapy, and the persistent challenge of HIV reservoirs and microenvironments, concluding with translational and ethical considerations essential for equitable global implementation.

## Lessons from Transplantation

The “Berlin Patient” provided the first clinical proof that durable HIV remission is possible [2]. After two allogeneic hematopoietic stem-cell transplants from a *CCR5Δ32* homozygous donor, Timothy Ray Brown achieved long-term viral remission without ART. Subsequent cases - the “London Patient” and the “New York Patient”- confirmed this principle, showing that reconstituting an HIV-resistant immune system can enable remission [3,4]. However, allogeneic transplantation carries major risks, including graft-versus-host disease and high treatment-related mortality [5]. Because *CCR5Δ32* donors are rare [6], this strategy is impractical for population-scale use. Nevertheless, these landmark cases redirected HIV-cure research toward engineering autologous hematopoietic stem/progenitor cells (HSPCs) capable of resisting infection—forming the foundation for gene-editing approaches.

## Gene Editing and Engineered Stem Cells

Genome-editing tools—Zinc-Finger Nucleases (ZFNs), Transcription Activator-Like Effector Nucleases (TALENs), and Clustered Regularly Interspaced Short Palindromic Repeats (CRISPR-Cas9) —allow precise disruption of HIV entry co-receptors such as CCR5 (C-C chemokine receptor type 5) in HSPCs. Clinical trials have shown safety and partial engraftment of CCR5-edited cells [7,8]. CRISPR-Cas9 offers superior efficiency and scalability but poses off-target risks that are being mitigated through high-fidelity Cas variants and prime-editing platforms [9]. Targeting CXCR4 is a complementary approach. Although CXCR4 editing can block X4-tropic strains, the receptor’s role in hematopoiesis and stem-cell homing prevents complete knockout [10]. Partial suppression or transient repression via CRISPR interference or small-molecule antagonists reduces viral entry while preserving hematopoietic function [11,12]. Induced pluripotent stem cells (iPSCs) extend gene-based therapies by providing renewable sources of HIV-resistant immune cells. CRISPR-modified iPSCs differentiated into T cells, macrophages, and NK cells have shown robust resistance in pre-clinical models [13]. The last two years have seen major progress: Fang *et al*. and Alidadi *et al*. outlined how iPSC-derived CAR-T (Chimeric Antigen Receptor T-cell therapy) and CAR-NK (Chimeric Antigen Receptor-Natural Killer cells) cells can be standardized for large-scale manufacturing and personalized therapy [14,15]. Concepts for universal/hypo-immunogenic donor lines are maturing [16]. Madrid *et al*. summarized translational and regulatory considerations for allogeneic iPSC-derived immune products [17]. Together these advances support an integrated strategy in which precise gene editing and stem-cell biology converge to create durable, HIV-resistant hematopoietic and immune systems.

## Stem-Cell and Gene-Editing Strategies for HIV Remission

Key gene-editing and stem-cell strategies under development for HIV remission are outlined in Table 1.

## Immunotherapy Integration

Stem-cell–based immunity can be enhanced by CAR-T and CAR-NK engineering. CAR-T cells targeting HIV Env or Gag have demonstrated safety but limited persistence [18]. Recent developments show that adoptively transferred mature CAR-T cells often exhibit exhaustion and fail to provide durable antiviral surveillance. Engineering hematopoietic stem/progenitor cells (HSPCs) to express CAR constructs can generate a renewable source of CAR-expressing effector cells that continually differentiate *in vivo* [19]. CAR-NK therapies provide complementary advantages, including MHC-independent recognition, lower graft-versus-host risk, and potent innate cytotoxicity [20]. Pilot trials using HSPC-derived CAR-NK cells are evaluating persistence, safety, and the potential to clear viral reservoirs in ART-suppressed individuals. Combining CAR-based immunity with latency-reversing agents (LRAs) could synergistically expose and eradicate latent HIV. This “shock-and-kill” paradigm, paired with gene-edited stem-cell–derived immunity, may convert transient remission into long-term functional cure [22].

## Reservoirs and Microenvironmental Challenges

Viral reservoirs persist in lymphoid tissues, the gut, and the central nervous system [22]. Within these niches, stromal interactions, cytokine gradients, and hypoxic conditions sustain latency and blunt immune clearance. Hypoxia modulates HIF-1α signaling, dampening antiviral immunity and supporting viral persistence [23]. Analogous to oncology, the HIV reservoir microenvironment can be therapeutically targeted. Agents modulating oxygen tension or cellular metabolism and 3-D organoid or tissue-chip systems now enable *ex vivo* testing of latency reversal and immune-clearance strategies [24]. These models provide physiologically relevant platforms to validate gene-edited immune interventions and guide translation to patients.

## Ethical and Regulatory Considerations

Gene editing for HIV cure raises ethical and safety concerns. Off-target mutagenesis, insertional oncogenesis, and long-term clonal drift remain the primary risks [9]. Current mitigation measures include high-fidelity Cas variants, base editing, and transient delivery vectors to limit nuclease exposure. The “CRISPR-baby” incident underscored the line between somatic and germline editing [25,26]. WHO and NIH bioethics frameworks [27,28] emphasize transparent governance, layered review, and community engagement. Equity must also remain central to implementation, as underscored by the UNAIDS 2024 report on fairness in the HIV response [29]. Equity must also remain central. Without deliberate access planning, advanced gene and cell therapies could widen global health disparities. Ensuring affordability, scalable manufacturing, and trial inclusion in high-burden regions will be critical to achieve a just HIV-cure landscape [29].

## Future Directions

Future HIV-cure paradigms will combine genetic resistance, immune reprogramming, and microenvironment modulation. We propose integrating (i) CCR5-edited autologous HSPCs, (ii) durable HSPC-derived CAR-T/NK cells, and (iii) LRAs to flush reservoirs, alongside oxygen- and metabolism-targeted adjuncts [18–23]. Developing universal hypo-immunogenic iPSC lines requires precise HLA editing (B2M/CIITA knockout with HLA-E/G retention) and strategies to avoid NK-cell “missing-self” rejection. Parallel efforts are refining differentiation protocols and engraftment durability [14–17]. Lessons from oncology—microenvironment targeting, combination immunotherapies, and adaptive trial design—can accelerate HIV translation. Precision-medicine frameworks incorporating viral tropism, HLA/KIR genotype, and reservoir profiling will personalize therapy and improve success rates.

## Conclusion

The *CCR5Δ32* transplantation cases proved that HIV cure is achievable. Advances in gene editing, stem-cell engineering, and immunotherapy now transform this insight into actionable strategies. The field’s success will depend on simultaneous progress in safety, microenvironment targeting, and equitable access. Stem-cell–based therapies are no longer experimental outliers—they form a cornerstone of the HIV-cure agenda aiming to make durable remission broadly attainable.

## Figures and Tables

**Table 1.** Summarizes the major gene-editing and stem-cell approaches currently under development for HIV remission.

Strategy	Target / Mechanism	Platform	Stage / Status	Key References
CCR5 gene knockout in HSPCs	Blocks HIV entry via CCR5 loss	ZFN / CRISPR	Phase I clinical (ongoing)	[7–9]
CXCR4 modulation	Partial suppression of CXCR4 expression	CRISPRi / antagonists / epigenetic repressors	Pre-clinical (2024)	[10–12]
iPSC-derived immune cells	Differentiation into HIV-resistant T, NK, or macrophage lineages	CRISPR-iPSC platforms	Pre-clinical (2024– 2025)	[13–17]
HSPC-derived CAR-T cells	Persistent anti-HIV adaptive immunity	Lentiviral CAR integration into HSPCs	NHP model and Phase I	[18,19]
HSPC-derived CAR-NK cells	Innate antiviral response with minimal GvHD risk	Retroviral CAR transduction of CD34^+^ cells	Pre-clinical and pilot trial (2025)	[20]
EBT-101 therapy	CRISPR-based excision of integrated HIV provirus	AAV-CRISPR (Excision Bio Therapeutics)	Phase I/II (2024)	[21]
